# Antioxidant Responses to Dietary Supplementation With Non‐Enriched and Selenium‐Enriched Mushrooms and Selenized Yeast in Piglets

**DOI:** 10.1002/mnfr.70609

**Published:** 2026-09-30

**Authors:** Diene France de de Souza, Gabriel Cipriano Rocha, Dairon Pérez Fuentes, Lucas Eduardo Oliveira Porto, Pedro Vitoriano Oliveira, Marliane de Cássia Soares da Silva, Maria Catarina Megumi Kasuya, Maiane de Paula Alves, Monique Renon Eller

**Affiliations:** ^1^ Department of Food Technology Universidade Federal de Viçosa (UFV) Viçosa Minas Gerais Brazil; ^2^ Department of Animal Science Universidade Federal de Viçosa (UFV) Viçosa Minas Gerais Brazil; ^3^ Institute of Chemistry Universidade de São Paulo (USP) São Paulo Brazil; ^4^ Department of Microbiology Universidade Federal de Viçosa (UFV) Viçosa Minas Gerais Brazil

**Keywords:** antioxidant activity, bioavailability, glutathione peroxidase, selenized mushroom, selenized yeast

## Abstract

Selenium plays an important role in antioxidant defense and metabolic regulation. This study evaluated the effects of dietary supplementation with non‐enriched and selenium‐enriched *Pleurotus ostreatus* mushrooms, as well as selenized yeast, on selenium deposition and antioxidant responses in piglets, a relevant model for human nutrition. Sixty‐four weaned piglets were fed control or supplemented diets for 21 days. Growth performance was not affected, indicating that the treatments were well tolerated. Selenium was higher in the loin of the supplemented groups than in the control group, whereas selenium concentration in the liver showed a numerical, but nonsignificant, increase in supplemented groups (*p* = 0.08), and no differences were observed in the kidney. Hepatic antioxidant activity was differentially modulated by dietary treatments. Glutathione peroxidase activity increased in all supplemented groups, whereas catalase activity increased only in piglets receiving selenium‐enriched mushroom or selenized yeast, and superoxide dismutase activity increased only in the selenium‐enriched mushroom group. Nitrite levels were reduced in all supplemented groups, while plasma antioxidant parameters remained unchanged. These findings indicate that dietary supplementation modulated hepatic antioxidant responses, although the relative contributions of selenium enrichment and mushroom bioactive compounds could not be determined.

**Trial Registration Number and Date of Registration**: 106/2018

## Graphical abstract

The findings highlight tissue‐specific responses to dietary selenium supplementation, with hepatic antioxidant modulation occurring without changes in plasma parameters, while selenium deposition patterns differed among tissues, emphasizing the complex relationship between selenium sources, metabolism, and antioxidant defense.

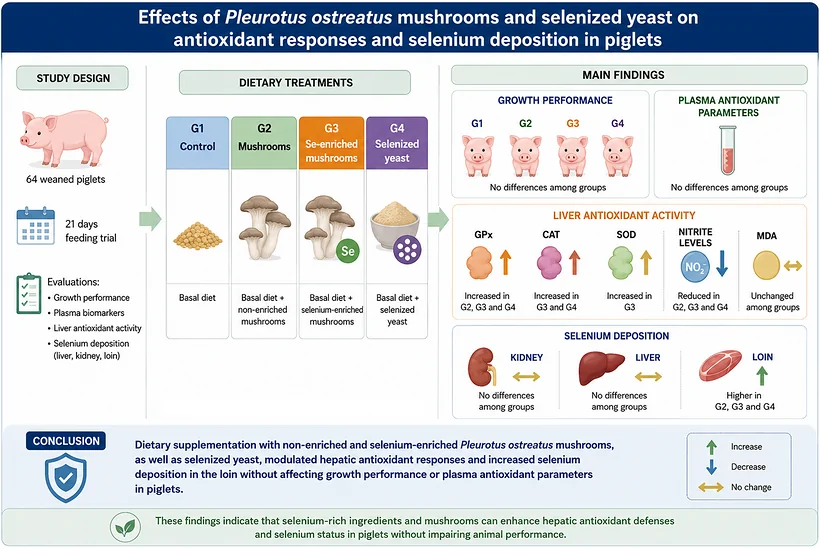

## 1 Introduction

Selenium is essential for maintaining physiological homeostasis, particularly by strengthening the immune system, supporting muscle regeneration, and contributing to reproductive function [1]. Many fundamental biological processes depend on selenium‐containing enzymes, such as glutathione peroxidase (GPx), whose active site contains selenocysteine. GPx converts reactive oxygen species (ROS)—natural by‐products of cellular metabolism—into less reactive molecules, thereby limiting oxidative damage [2, 3] (Figure 1).

**FIGURE 1:**
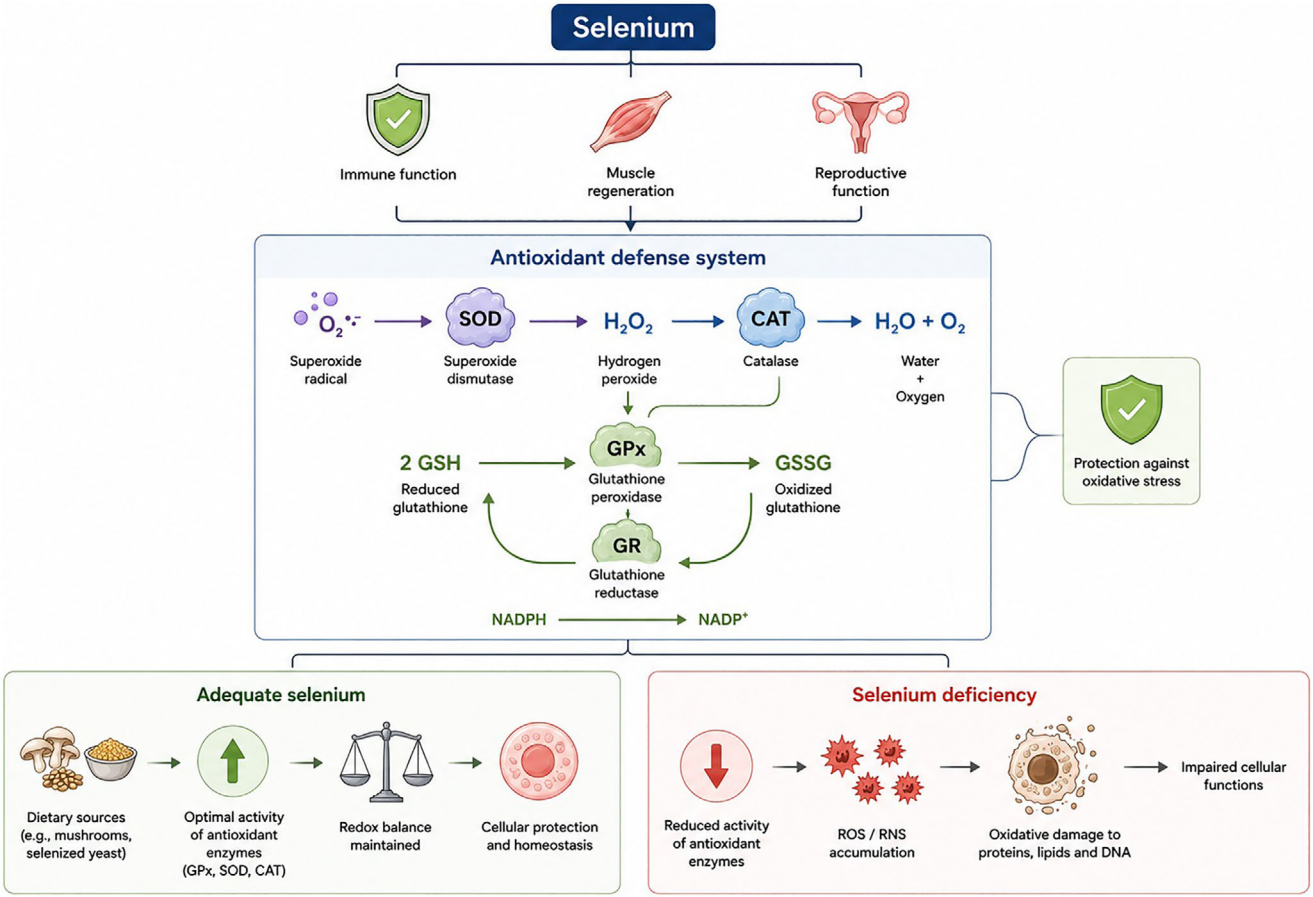
Graphical overview of the biological role of selenium in antioxidant defense. Selenium supports antioxidant defense through the coordinated activity of SOD, CAT, GPx, and GR, maintaining cellular redox homeostasis. Adequate selenium intake promotes antioxidant protection, whereas selenium deficiency impairs antioxidant defenses, increases oxidative stress, and leads to cellular damage.

In addition to GPx, the endogenous antioxidant system includes superoxide dismutase (SOD), responsible for the formation of peroxides from the superoxide radical (O^−·^_2_), and catalase (CAT), which subsequently transform these peroxides into water and oxygen, thereby counteracting oxidative stress [4]. Notably, the catalytic activity of GPx reduces hydrogen peroxide by oxidizing GSH to oxidized‐glutathione (GSSG), which is subsequently regenerated by glutathione reductase (GR) using NADPH as a cofactor [5]. The presence of selenium in the active site of selenium‐dependent GPx amplifies its enzymatic activity. Consequently, selenium consumption can lead to an augmentation of this enzyme's function, thereby influencing the coordinated responses of CAT and SOD, as these enzymes collaborate to combat oxidative stress [6].

Conversely, in situations where selenium is limited, the diminished activity of antioxidant enzymes can lead to an accumulation of ROS and reactive nitrogen species (RNS)—including malondialdehyde (MDA), an end‐product of lipid peroxidation—along with nitric oxide (NO) [7]. Because NO is highly unstable, it is rapidly oxidized to nitrite (NO_2_^−^) and nitrate (NO_3_^−^), which are commonly used as indirect biomarkers of NO production. Low concentrations of NO play a protective role in the organism by mitigating ischemic damage and promoting blood circulation, whereas elevated levels are correlated with adverse outcomes such as toxicity, inflammation, and, in more severe instances, septic shock [8]. Selenium deficiency has also been linked to conditions such as Keshan disease (cardiomyopathy) and Kashin–Beck disease (endemic osteoarthritis) [9].

In certain situations, selenium supplementation becomes necessary. This can be achieved through inorganic (selenite, selenate) or organic (selenomethionine, selenocysteine) forms of selenium [10]. Another promising strategy involves consuming selenium‐enriched foods, such as edible mushrooms. Edible mushrooms can absorb minerals and incorporate them into their fruiting bodies [11, 12, 13], thereby converting inorganic selenium into more bioavailable organic forms [14]. Similarly, yeasts, such as *Saccharomyces cerevisiae*, can also perform this conversion and are widely used in animal nutrition [15, 16]. Despite this, there is a lack of comparative in vivo studies evaluating which of these selenium‐enriched foods offers higher bioavailability and greater potential to improve health and performance in humans or animals.

Previous research has shown that consuming selenium‐enriched mushroom increases plasma selenium levels [11] and enhances GPx activity in the colons of rats [17]. In diabetic mice, these mushrooms also increased GPx activity in the liver and kidneys [18]. Comparative in vivo studies evaluating different selenium sources, such as selenium‐enriched mushroom and selenized yeast, remain limited, especially in piglets, which are considered a relevant model for human nutrition due to their physiological similarities [19]. Moreover, the potential contribution of mushrooms, independent of selenium enrichment, has not been fully elucidated.

Therefore, the objective of this study was to evaluate the effects of dietary supplementation with non‐enriched and selenium‐enriched *Pleurotus ostreatus* mushrooms, as well as selenized yeast, on selenium deposition in tissues (kidney, liver, and loin) and antioxidant responses in plasma and liver of piglets.

## 2 Experimental Section

### 2.1 Experimental Design

The experiment was approved by the Ethics Committee on the Use of Production Animals—CEUAP/UFV, process number 106/2018, and conducted at the experimental pig farm of the Department of Animal Science, at Universidade Federal de Viçosa (UFV).

Sixty‐four AGPIC 415 × Camborough piglets (Agroceres PIC, MG, Brazil), weaned at 21 days of age with an initial weight of 7.25 ± 0.64 kg, were allocated to treatments using a randomized complete block design based on initial body weight (Figure 2). Piglets were classified into two weight blocks (light: 6.52 ± 0.23 kg; heavy: 7.70 ± 0.29 kg) before random assignment to the dietary treatments. Each treatment was represented by three pens in the light block and five pens in the heavy block. Block was included as a factor in the analysis of variance. Over a period of 21 days, the piglets were allocated to four treatment groups:

- Group 1 (G1) received the control diet.
- Group 2 (G2) was fed a diet containing non‐enriched mushroom.
- Group 3 (G3) received a diet containing selenium‐enriched mushroom.
- Group 4 (G4) was provided with a diet containing selenized yeast.

**FIGURE 2:**
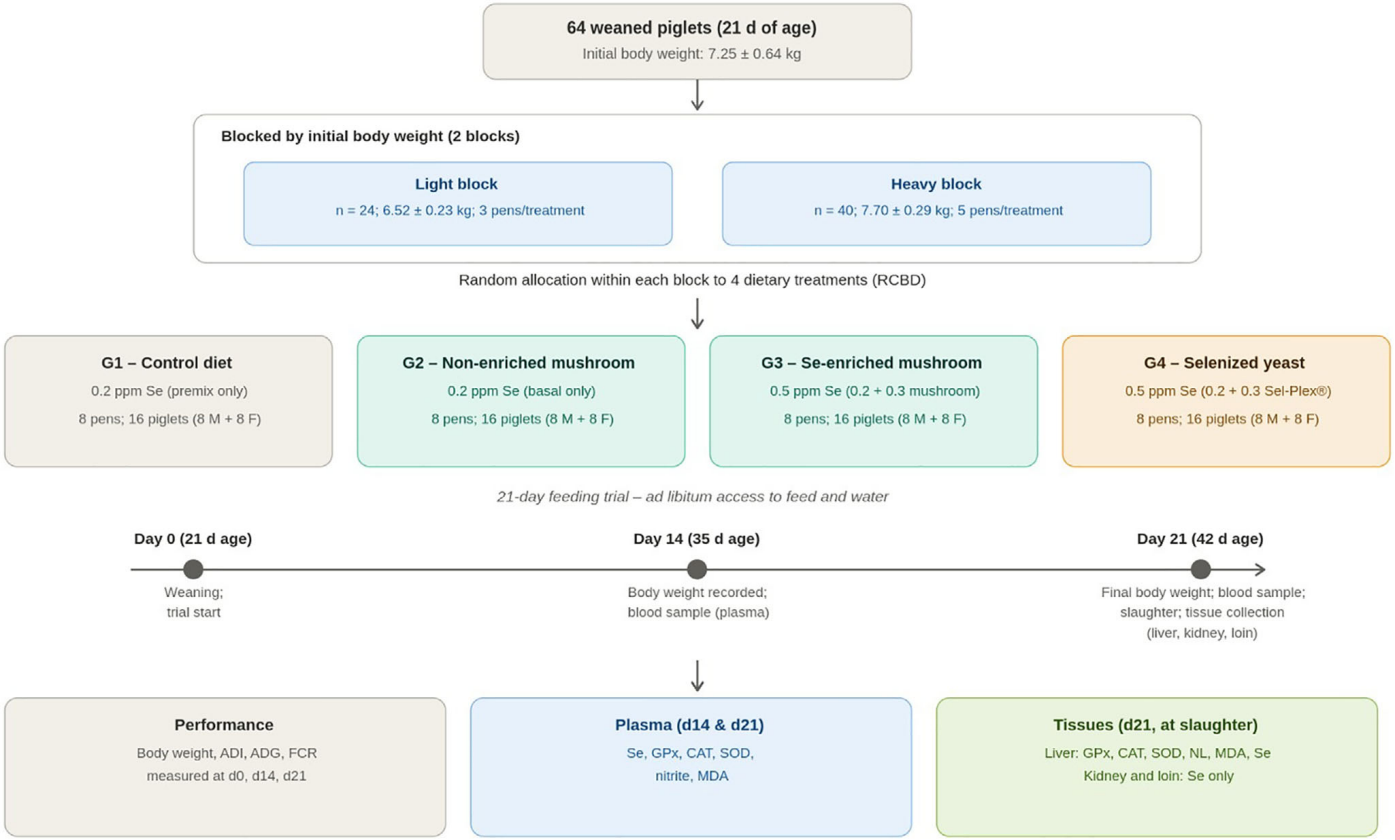
Schematic representation of the experimental design. Sixty‐four weaned piglets were assigned to a randomized complete block design based on initial body weight and allocated to four dietary treatments (control, non‐enriched mushroom, selenium‐enriched mushroom, or selenized yeast) for 21 days. Body weight was recorded on days 0, 14, and 21; blood samples were collected on days 14 and 21; and liver, kidney, and loin samples were collected at slaughter (day 21).

Each treatment consisted of eight experimental units (pens), with two piglets (one male and one female) housed per pen. The temperature within the experimental room ranged from 25.5°C to 28.0°C. Water and feed were provided ad libitum.

### 2.2 Mushrooms Production

The cultivation of mushrooms followed the method outlined by Souza et al. (2021) [13], with adaptations. Briefly, the *P*. *ostreatus* (PLO 02) isolate was obtained from a fungal collection of the Laboratory of Mycorrhizal Associations, Department of Microbiology/BIOAGRO, at UFV, Minas Gerais, Brazil. This isolate was cultured in a Petri dish containing potato dextrose agar (PDA, Merck, Darmstadt, Germany), pH 5.8, and incubated in a biochemical oxygen demand (BOD) incubator at 25°C for seven days. Subsequently, four mycelial discs were transferred to 100 g of sorghum grains for spawn production. These grains were precooked for 40 min and autoclaved at 121°C for one hour. Sugarcane bagasse served as the substrate for mushroom cultivation. For this, the byproduct was dried, fragmented into approximately 2 cm pieces, and soaked in a hydrated lime solution (Ca(OH)_2_) at a concentration of 2% (w/v) for 16 h. Afterward, centrifugation (Britânia, 37501002) for 1 min at 600 rpm (50.4 g) was carried out to eliminate excess lime solution. Following this, 1 kg of the prepared substrate was placed into polypropylene bags. In each bag, 10 mL of either distilled water or a sodium selenite solution, containing 25 mg kg^−1^ of selenium, was introduced. The spawn was then inoculated, and the bags were sealed and incubated at 25°C until the mycelia completely colonized the substrate. Subsequently, a thermal shock through a temperature drop (to 10°C) was implemented for 24 h to induce fruiting. The bags were kept at 23°C and 80% relative humidity until the mushrooms were harvested at a stage where their caps were facing downward. The harvested mushrooms were dehydrated using a food dehydrator at 55°C (Meloni, Pratic Dryer) until a constant weight was achieved. Following dehydration, the mushrooms were ground using a mini food processor (Palcookin, TK102).

### 2.3 Feed Preparation

The piglets' basic diet consisted of a blend of vitamin‐mineral premix with corn and soybean meal (G1), formulated as a mash with 3400 kcal kg^−1^ of metabolizable energy (ME) and 14.5 g kg^−1^ of digestible lysine. The selenium content of this premix is 212.5 µg per kg (Table 1), or approximately 0.2 ppm. Following the ideal amino acid profile recommended by the Brazilian Tables for Poultry and Swine [20], the minimum proportions of digestible methionine + cysteine, threonine, tryptophan, and valine were set at 56%, 63%, 18%, and 69% of digestible lysine, respectively. The selenized yeast (Sel‐plex) and selenium‐enriched mushroom powder used for feed supplementation were maintained in a desiccator for later use in preparing the feed. These products were quantified for selenium content following the methodology outlined in Souza et al. (2021) [13]. The quantities of these products in the feed were determined based on their selenium concentrations, which were 1000.00 and 196.00 µg g^−1^, respectively. The values were chosen to adhere to the Food and Drug Administration's recommendation of 0.3 ppm of selenium supplementation [21]. Thus, in G2 and G3, corn was substituted with 1.53 g kg^−1^ of mushroom powder or selenium‐enriched mushroom powder, respectively. For G4, the diets included 0.3 g kg^−1^ of the selenized yeast Sel‐plex (Alltech Inc., USA). Sel‐plex was selected due to its established use in animal production for this purpose. It comprises an extract of *S. cerevisiae* cells cultivated on a medium enriched with sodium selenite [22]. The formulations of diets fed to the piglets are demonstrated in Table 1. The total selenium content calculated for groups G1 and G2 is 0.2 ppm (from premix), and for groups G3 and G4 is 0.5 ppm, that is, 0.2 ppm from the premix and 0.3 ppm from the selenium source. Selenium concentrations were calculated based on the analyzed selenium contents of the supplemented ingredients and the diet formulation and were not analytically determined in the complete diets.

**TABLE 1:** Formulations of diets fed to the piglets.

	Treatment			
Ingredients	G1	G2	G3	G4
Vitamin‐mineral premix (kg)	50.00	50.00	50.00	50.00
Corn meal (kg)	86.00	85.694	85.694	85.94
Soybean meal (kg)	54.00	54.00	54.00	54.00
Saccharose (kg)	10.00	10.00	10.00	10.00
Mushroom powder (kg)	0.00	0.306	0.00	0.00
Selenized mushroom powder (kg)	0.00	0.00	0.306	0.00
Selenized yeast	0.00	0.00	0.00	0.06
Total dietary selenium (ppm)	0.20	0.20	0.50	0.50
Total Weight (kg)	200.00	200.00	200.00	200.00

*Note*: G1, control diet; G2, diet with non‐enriched mushroom; G3, diet with selenium‐enriched mushroom; G4, diet with selenized yeast. Vitamin‐mineral premix provided the following per kilogram of complete diet: vitamin A, 16 000 IU; vitamin D3, 2,850 IU; vitamin E, 40 IU; vitamin K, 6 mg; thiamine, 4,750 µg; riboflavin, 16 mg; pyridoxine, 2750 µg; vitamin B12, 47.5 µg; folic acid, 750 µg; biotin, 250 µg; pantothenic acid, 32 mg; niacin, 60 mg; copper, 100 mg; iodine, 1000 µg; iron, 88 mg; manganese, 35 mg; selenium, 212.5 µg; zinc, 1800 mg.

### 2.4 Performance

Growth performance was evaluated by determining the average weight of both animals within each pen (repetition). For this, individual weighing of the weaned piglets took place at three points: 21 days old (day 0 of the experiment), 35 days old, and 42 days old (day 21 of the experiment). Concurrently, feed quantities, leftovers, and waste were measured within the same time frame. This data was employed for calculating the following parameters:

1. Average daily intake (ADI) in kg/day: This represents the daily amount of feed consumed by the animals.
2. Average daily gain (ADG) in kg/day: denotes the daily increase in weight of the animals.
3. Feed conversion ratio (FCR = ADI/ADG): indicates the efficiency of feed utilization, relating to the amount of feed consumed to the weight gained.

### 2.5 Sample Collection and Preparation

Blood samples of 5 mL each were collected from one piglet per pen by puncturing the orbital sinus at both 35 and 42 days old, with the material collected kept in heparin tubes. The collected blood was promptly refrigerated, and plasma was obtained through centrifugation at 3,584 g for 10 min, followed by storage at ‐20°C. At the end of the experimental period, after 12 h fasting, one piglet per pen was humanely slaughtered. The piglet was rendered unconscious using head‐only electrical stunning (240 V, 1.3 A). Immediately following the exsanguination process, samples of the kidney, liver, and loin were extracted and stored at ‐80°C.

For assessing the antioxidant activity of the liver, 100 mg of tissue pieces were homogenized in 1 mL of 100 mM phosphate buffer (pH 7.4). The homogenate was centrifuged at 3,500 g and 4°C for 10 min, and 1 mL of the supernatant was retrieved and stored at ‐20°C. The kidney, loin, and a portion of the liver were lyophilized, then ground using a miniprocessor (Palcookin, TK102), and subsequently sieved for selenium analysis. Plasma samples were directly employed for antioxidant analyses, which were conducted using microplates. The absorbance readings were performed using a Multiskan GO (Thermo Scientific) spectrophotometer.

### 2.6 Determination of GPx, CAT and SOD Activities in Plasma and in Liver

The activities of GPx, CAT, and SOD were expressed as U mL^−1^ (plasma) or U mg protein^−1^ (liver). The total protein content was determined using the Bradford method [23].

The GPx activity was determined following the procedure outlined by Cichoski et al. [24], with slight adjustments. Briefly, 15 µL of plasma or 5 µL of liver homogenate were dissolved in 10 µL of a phosphate buffer (60 mM) solution containing sodium azide (1.0 mM) and EDTA (0.5 mM) at pH 7.0 (reaction buffer). Sodium azide was used to inhibit CAT activity [25]. As a control, 15 µL of the reaction buffer was used. The reaction mixture comprised 243 µL of reaction buffer with GSH (1.12 mM), NADPH (0.2 mM), and GR (0.243 U).

Initiating the reaction involved the addition of 42 µL of H_2_O_2_ at 0.72 mM. Following a 10 min incubation at 37°C, the reduction in NADPH absorbance was monitored at 340 nm for 5 min. One unit of GPx activity was defined as the quantity of enzyme necessary to oxidize 1 nM of NADPH to NADP**^+^** per minute, and was calculated using Equation (1):

(1) $$U=\frac{\left[\frac{\left(\Delta Aa-\Delta Ac\right)}{t}*F\right]}{Q}\;$$

where ∆Aa and ∆Ac represent the reduction in absorbances for samples and controls, respectively; *t* is the reaction duration, in minutes; *Q* signifies the protein content in the liver (mg); and *F* is a constant utilized to convert absorbance per minute (∆*A*/*t*) into enzyme units (U). The value of *F* is derived through the following Equation (2):

(2) $$F=\left[\frac{\frac{Vr}{Va}}{0.00373}\right]\;$$

where Vr stands for the reaction volume, Va denotes the sample volume, and 0.00373 is the molar extinction coefficient (µM^−1^ cm^−1^), calculated considering the current NADPH molar extinction coefficient (0.00622 µM^−1^ cm^−1^) and the optical path the light takes on a microplate (0.6 cm) [26].

The CAT activity was assessed by analyzing the kinetics of hydrogen peroxide (H_2_O_2_) decomposition, following the methodology outlined by Aebi [27]. The SOD activity was estimated using the pyrogallol method, which relies on the enzyme's capacity to catalyze the reaction between superoxide (O^−·^_2_) and hydrogen peroxide (H_2_O_2_) [28].

### 2.7 Determination of MDA and Nitrite Contents in Plasma and in Liver

The MDA content was determined according to the procedure established by Buege and Aust (1978) [29]. For this analysis, 400 µL of a thiobarbituric acid solution comprising trichloroacetic acid at 15% w/v, thiobarbituric acid at 0.375% w/v, and HCl at 0.25 N was combined with 200 µL of plasma or liver homogenate. The reaction was conducted in a water bath at 90°C for 40 min, and then the microtubes were allowed to reach room temperature for equilibrium. Subsequently, 600 µL of butyl alcohol (P.A.) were added to the microtubes, which were then subjected to centrifugation at 1200 *g* for 5 min at room temperature. The supernatant was utilized for absorbance readings. The formation of thiobarbituric acid reactive substances was monitored at 535 nm. The MDA content, expressed in mg of protein, was calculated as the ratio of the absorbance and the molar extinction coefficient of thiobarbituric acid (*Ɛ* = 0.156 µmol L^−1^).

The nitrite levels (NL) were measured by quantifying nitrites via Griess' standard reaction. In brief, 50 µL of plasma or liver homogenate were incubated with an equal volume of Griess’ solution (sulfanilamide 1% w/v, naphthyl ethylenediamine dichlorhydrate 0.1% w/v, and phosphoric acid 2.5% v/v) for 10 min. Absorbance was measured at 540 nm. NL were calculated using a sodium nitrite (NaNO_2_) standard curve spanning from 0 to 100 µM [30]. NO was not directly measured, and NL were used as an indirect marker of NO.

### 2.8 Selenium Concentration Analysis

#### 2.8.1 Sample Digestion

Selenium concentration was determined in plasma, beyond the lyophilized samples (liver, kidney, and loin).

Ten grams of the lyophilized samples were subjected to further grinding in a cryogenic mill (Marconi, Brazil) equipped with a self‐contained liquid nitrogen bath. After 5 min of pre‐cooling, the grinding process comprised 5 cycles, with each cycle consisting of 2 min of grinding followed by a 1 min cooling stage. Next, 200 mg was subjected to digestion in a microwave oven (Anton Paar, Multiwave‐3000) using a diluted acid mixture comprising 3.0 mL nitric acid (HNO_3_), 1.0 mL hydrogen peroxide (H_2_O_2_), and 2.0 mL H_2_O. The digestion occurred in closed vessels and was performed through four steps of temperature, ramp rate, and hold time, respectively: i) 140°C, 5 min, 1 min; ii) 180°C, 4 min, 5 min; iii) 200°C, 4 min, 10 min; and iv) 0°C, 0 min, 20 min.

#### 2.8.2 Selenium Determination

The selenium determination was carried out using a graphite furnace atomic absorption spectrometer (Analytik Jena AG, ZEEnit 60), equipped with an autosampler, a transversely heated graphite atomizer, pyrolytically coated graphite tube, and transversal Zeeman‐effect background corrector. For selenium determination, 10 µL of chemical modifier (5 µg of Pd + 3 µg of Mg (prepared from Pd (NO_3_)_2_ and Mg (NO_3_)_2_, both from Merck) was co‐injected into the graphite furnace with 10 µL of samples or analytical calibration solutions (5 to 90 µg L^−1^ in 1.0 M HNO_3_). A Titrisol standard solution of 1000 mg L^−1^ of Se (Merck) was used to prepare the reference analytical solutions in 0.14 M HNO_3_. For selenium determination in plasma, a reference solution containing 50.0 µg L^−1^ of Se in 0.1% w/v Triton X‐100 + 1.0% v/v HNO_3_ and a chemical modifier of 5 µg of Pd + 3 µg of Mg in 1.0% v/v HNO_3_ was used. The analytical reference solutions for the spectrometer calibration were prepared directly in the autosampler cups (total volume = 1200 µL), diluting 300 µL of the stock analytical reference solutions containing 20, 40, 80, 160, 320 µg L^−1^ of Se with 900 µL of 0.15% w/v Triton X‐100 + 1.5% v/v HNO_3_. For samples, an aliquot of 30 µL of plasma sample was transferred into the autosampler cup containing 90 µL of 0.15% w/v Triton X‐100 + 1.5% v/v HNO_3_. The resulting dispersion was mixed by pumping the micropipette several times in the autosampler cup to ensure that the plasma constituents were completely lysed. Argon 99.998% (v^−1^) (Air Liquide Brasil, Brazil) was used as protective and purge gas. The heating program for graphite furnace used for all samples was executed as follow (step, temperature/°C, ramp/s, hold/s): (drying I, 80, 5, 10); (drying II, 130, 20, 5); (pyrolysis I, 400, 10, 15); (pyrolysis II, 1200, 100, 30); (atomization, 2300, 0, 5); and (cleaning 2500, 500, 2). In all steps, argon flow rate was 250 mL min^−1^, except during atomization that was interrupted [31]. The results were expressed as micrograms of selenium per gram of organs and tissue or per liter of plasma.

### 2.9 Statistical Analysis

The statistical analysis was conducted using the R software (version 3.6.2). First, the results underwent a normality test using the Shapiro‐Wilk test to assess the variance distribution. If the data did not meet the criteria for normality, the Box & Cox transformation method was applied [32]. After the new statistical analysis, the data were reconverted to their original values.

An analysis of variance (ANOVA) was performed using the F test, with a significance level set at *p* < 0.05, considering treatment and block as factors. For plasma variables, the day of blood collection (35 and 42 days of age) was also included in the model to account for repeated measurements over time. Statistical significance was declared at *p* < 0.05, and when significant effects were detected, means were compared using Tukey's test (*p* < 0.05).

Selenium concentration was determined in all plasma, kidney, and loin samples (*n* = 8 per treatment). For liver samples, selenium concentration was determined in five samples per treatment (*n* = 5), because some liver samples were lost during laboratory analysis.

## 3 Results

### 3.1 Performance

Feeding was introduced on the second day of the experiment, a trend supported by previous findings [33]. There was no difference (*p*>0.05) in the ADG (kg day^−1^), ADI (kg day^−1^), and FCR (ADI ADG^−1^), regardless of the feed composition provided for each group (Table 2), indicating that the inclusion of mushrooms, selenized mushrooms, or selenized yeast in the feed did not influence the performance of animal production.

**TABLE 2:** Performance of piglets fed with different sources of organic selenium.

Parameter	Treatment				CV	*p*
	G1	G2	G3	G4		
BW – 21 d (kg)	7.23 ± 0.60	7.28 ± 0.73	7.26 ± 0.58	7.26 ± 0.66	2.84	0.97
BW – 35 d (kg)	10.01 ± 1.09	10.25 ± 1.09	9.77 ± 1.14	10.43 ± 1.00	7.92	0.39
BW – 42 d (kg)	13.15 ± 1.31	13.43 ± 1.38	13.18 ± 1.68	13.65 ± 1.22	8.28	0.78
21‐35 d						
ADI (kg)	0.26 ± 0.07	0.28 ± 0.04	0.26 ± 0.03	0.28 ± 0.04	17.67	0.69
ADG (kg)	0.21 ± 0.06	0.21 ± 0.03	0.20 ± 0.03	0.23 ± 0.05	21.74	0.63
FCR	1.29 ± 0.08	1.32 ± 0.08	1.32 ± 0.08	1.29 ± 0.09	6.56	0.89
21‐42 d						
ADI (kg)	0.37 ± 0.08	0.39 ± 0.05	0.37 ± 0.04	0.39 ± 0.05	14.52	0.83
ADG (kg)	0.28 ± 0.06	0.29 ± 0.04	0.30 ± 0.03	0.30 ± 0.05	14.96	0.76
FCR	1.32 ± 0.06	1.33 ± 0.02	1.29 ± 0.11	1.29 ± 0.06	5.27	0.70

*Note*: G1, control diet; G2, diet with non‐enriched mushroom; G3, diet with selenium‐enriched mushroom; G4, diet with selenized yeast; CV, coefficient of variation; *p*, *p*‐value; BW, body weight; d, days; ADI, average daily intake; ADG, average daily gain; FCR, feed conversion ratio. For each parameter analyzed, the averages did not differ by *F* test (*p* > 0.05).

### 3.2 Selenium and Antioxidant Activity in Plasma

The control diet (G1) as well as diets supplemented with non‐enriched mushroom (G2), selenium‐enriched mushroom (G3), or selenized yeast (G4) showed no significant differences in plasma selenium concentrations and antioxidant activity, with no significant differences observed among treatments (*p*>0.05) (Table 3).

**TABLE 3:** Selenium content, activity of enzymes GPx, CAT, and SOD, and NL and MDA contents in the plasma of piglets.

Parameter	Treatment				CV	*p*
	G1	G2	G3	G4		
35 d						
Se (µg L^−1^)	17.88 ± 4.50	14.51 ± 9.15	20.69 ± 9.55	19.24 ± 5.86	42.49	0.43
GPx (U mL^−1^)	67.96 ± 6.77	70.91 ± 4.36	63.27 ± 9.23	66.62 ± 8.97	11.50	0.28
Cat (U mL^−1^)	185.6 ± 48.75	200.55 ± 29.58	191.41 ± 53.05	175.74 ± 11.44	18.60	0.56
SOD (U mL^−1^)	4.17 ± 2.02	3.05 ± 2.58	4.31 ± 1.39	3.78 ± 3.10	62.73	0.73
NL (µM)	8.70 ± 1.10	9.17 ± 1.08	8.53 ± 0.69	7.86 ± 0.97	11.54	0.09
MDA (nM mg prot^−1^)	0.34 ± 0.18	0.24 ± 0.05	0.27 ± 0.22	0.29 ± 0.13	55.48	0.68
42 d						
Se (µg L^−1^)	20.88 ± 6.80	19.42 ± 7.45	20.41 ± 11.06	19.85 ± 5.92	37.53	0.98
GPx (U mL^−1^)	76.94 ± 5.28	79.09 ± 5.15	75.74 ± 8.07	76.54 ± 5.72	8.15	0.74
CAT (U mL^−1^)	148.39 ± 32.57	170.39 ± 58.23	162.00 ± 36.16	159.81 ± 37.82	26.39	0.78
SOD (U mL^−1^)	3.75 ± 2.60	3.21 ± 1.78	2.81 ± 2.22	4.07 ± 1.47	60.45	0.64
NL (µM)	8.67 ± 2.42	8.11 ± 1.44	8.13 ± 1.63	7.13 ± 0.59	19.52	0.28
MDA (nM mg prot^−1^)	0.30 ± 0.27	0.23 ± 0.15	0.18 ± 0.08	0.19 ± 0.06	73.27	0.44

*Note*: G1, control diet; G2, diet with non‐enriched mushroom; G3, diet with selenium‐enriched mushroom; G4, diet with selenized yeast; CV, coefficient of variation; *p*, *p*‐value; prot, protein. For each parameter analyzed, averages do not differ significantly by *F* test (*p* > 0.05).

### 3.3 Antioxidant Activity and Se Concentration in Liver

Regarding selenium accumulation in the liver, no statistically significant differences were observed among treatments (*p* = 0.08), although numerically higher values were noted in animals receiving supplemented diets compared to the control group (G1) (Table 4). These results indicate that, under the conditions evaluated, dietary supplementation did not result in a measurable increase in hepatic selenium deposition.

**TABLE 4:** Activity of enzymes GPx, CAT, and SOD, and NL and MDA contents in the liver of piglets, and selenium accumulation in tissues.

	Treatment					
Parameter	G1	G2	G3	G4	CV	*p*
GPx in liver (U mg prot^−1^)	14.55 ± 3.31 ^b^	23.29 ± 6.49 ^a^	26.51 ± 8.42 ^a^	23.17 ± 4.93 ^a^	28.31	0.005
CAT in liver (U mg prot^−1^)	16.18 ± 3.90 ^b^	23.27 ± 4.55 ^ab^	29.28 ± 12.35 ^a^	26.22 ± 4.96 ^a^	30.41	0.008
SOD in liver (U mg prot^−1^)	1.06 ± 0.18 ^b^	1.56 ± 0.38 ^ab^	1.80 ± 0.67 ^a^	1.54 ± 0.31 ^ab^	28.73	0.014
NL in liver (µM)	11.98 ± 3.97 ^a^	7.67 ± 1.13 ^b^	7.56 ± 1.02 ^b^	7.45 ± 1.72 ^b^	25.51	0.002
MDA in liver (nM mg prot^−1^)	0.43 ± 0.09 ^a^	0.31 ± 0.09 ^a^	0.43 ± 0.18 ^a^	0.39 ± 0.14 ^a^	33.65	0.246
Se in liver (µg g^−1^)	0.87 ± 0.27 ^a^	1.03 ± 0.25 ^a^	1.27 ± 0.16 ^a^	1.33 ± 0.36 ^a^	24.04	0.08
Se in loin (µg g^−1^)	0.54 ± 0.16 ^b^	0.97 ± 0.23 ^a^	0.78 ± 0.35 ^a^	0.80 ± 0.29 ^a^	35.54	0.04
Se in kidney (µg g^−1^)	2.76 ± 0.69 ^a^	2.30 ± 0.63 ^a^	2.67 ± 0.76 ^a^	2.98 ± 0.75 ^a^	26.95	0.37

*Note*: G1, control diet; G2, diet with non‐enriched mushroom; G3, diet with selenium‐enriched mushroom; G4, diet with selenized yeast; CV, coefficient of variation; *p*, *p*‐value; prot, protein. For each parameter analyzed, averages with different letters differ significantly by Tukey's test (*p* < 0.05).

Hepatic antioxidant activity was differentially affected by the dietary treatments (Table 4). Compared with the control group (G1), GPx activity was significantly higher in G2, G3, and G4 (*p* < 0.05). In contrast, CAT activity was significantly increased only in G3 and G4, whereas SOD activity was significantly higher only in G3.

Furthermore, a significant reduction in NL was observed in the liver of piglets from all supplemented groups compared with G1. In contrast, MDA levels remained unaffected across all treatments (*p*>0.05), suggesting that baseline lipid peroxidation was unaltered despite the enzymatic modulation.

### 3.4 Selenium Accumulation in Tissues

Regardless of the treatment, selenium accumulation was highest in the kidney, followed by the liver, and lowest in the loin (Table 4).

In the loin, selenium concentration was significantly higher in G2, G3, and G4 than in the control group (G1) (*p* < 0.05) (Table 4).

In contrast, no significant effects of dietary treatments were observed on selenium concentration in the kidney or liver (*p*>0.05) (Table 4), despite numerically higher values in the supplemented groups.

## 4 Discussion

### 4.1 Performance

The lack of differences in ADG, ADI, and FCR indicates that the inclusion of mushrooms or selenized yeast did not influence production performance; this outcome is noteworthy as it underscores the palatability of mushrooms to the animals and their overall well‐being. Interestingly, the introduction of other organic forms of selenium, such as selenomethionine [34] and a selenium‐hydroxy‐methionine analogue [35], similarly showed no performance effects on piglets. Comparable results were observed in rats fed with selenium‐rich spirulina, selenomethionine [36], and selenium‐enriched *Agaricus bisporus* mushroom [37]. This collective evidence highlights that selenium‐enriched dietary components did not impair animal performance, supporting the safety of these supplementation strategies.

### 4.2 Selenium and Antioxidant Activity in Plasma

The maintenance of enzyme activities and selenium content in the plasma of the animals suggests that the diets were not harmful to the animals. Moreover, it indicates that the selenium from both the mushrooms and yeast supplements was effectively metabolized by the liver and either excreted or transported to other sites in the body, such as the kidney and loin, since organic selenium follows different routes and can even be randomly incorporated to replace methionine during the protein synthesis process [22]. No significant differences were observed among treatments for plasma selenium concentration or antioxidant parameters, indicating that the dietary interventions did not measurably affect systemic oxidative status under the conditions of the present study. These findings suggest that the effects of these dietary interventions were more evident at the tissue level than in systemic circulation.

### 4.3 Antioxidant Activity and Se Concentration in Liver

Although numerically higher liver selenium concentrations were observed in the supplemented groups, no statistically significant differences were detected among treatments. Therefore, under the conditions of the present study, dietary supplementation did not result in a measurable increase in hepatic selenium deposition.

The numerical increase observed in G2, despite receiving the same calculated selenium concentration as the control diet, should be interpreted with caution. Although mushroom bioactive compounds may influence mineral absorption or metabolism, this hypothesis was not directly evaluated in the present study. Biological variability, sample size, and the possibility of a chance finding cannot be excluded.

Hepatic antioxidant activity was modulated by the dietary treatments; however, the response depended on the biomarker evaluated. GPx activity increased significantly in all supplemented groups, whereas CAT activity increased only in G3 and G4, and SOD activity increased only in G3. In contrast, liver NL was reduced in all supplemented groups, while MDA levels remained unchanged.

Mushrooms contain bioactive compounds such as phenolics and β‐glucans that have been associated with antioxidant effects [4]. Studies with elderly rats receiving *P. ostreatus* extract showed increased CAT expression and glutathione‐system enzyme activity [45, 46, 47]; consumption of this mushroom has also been associated with reductions in hypercholesterolemia and hyperglycemia in various animal models [48, 49, 50]. The increases in hepatic antioxidant activity in groups G3 and G4 may be associated with selenium supplementation and/or phytochemicals present in mushrooms and yeast [13]. For instance, dietary selenized yeast at 0.2 ppm Se increased hepatic GPx in broiler chicks [16], and at 0.5 ppm reduced liver NO in cadmium‐contaminated chickens [51]. Other Se‐enriched sources similarly elevated hepatic antioxidant markers in different models [52, 53].

Collectively, these findings suggest that both selenium supplementation and mushroom‐derived bioactive compounds may have contributed to the observed antioxidant responses. However, because the present experimental design did not include a non‐selenized yeast control and was not intended to test interactions between selenium and mushroom constituents, the relative contribution of each component could not be determined.

### 4.4 Selenium Accumulation in Tissues

The increase in selenium concentration observed in the loin of groups G2, G3, and G4 (*p*<0.05), including the response in the group receiving non‐selenized mushrooms (G2), suggests that dietary supplementation was associated with higher selenium concentration in this tissue. Although G2 received no additional selenium beyond the basal diet, the observed increase in loin selenium concentration should be interpreted with caution. Biological variability, sample size, and the possibility of a chance finding cannot be excluded. Therefore, the mechanism underlying this response remains uncertain.

In the kidney, lack of treatment effect aligns with other studies involving growing–finishing pigs that received selenium‐enriched diets and showed similar kidney Se content (0.66–0.70 µg g^−^^1^) across treatments (0.6%–2.4% of selenized *Auricularia cornea*) [38]. This consistent accumulation trend aligns with observations from other studies involving piglets, carp, and rats supplemented with organic selenium sources [38, 39, 40, 41].

However, in contrast, research involving broilers and mice that received selenium supplementation demonstrated greater selenium accumulation in the liver compared to the kidney [42, 43]; this divergence indicates that selenium accumulation patterns vary by species. It is noteworthy that these patterns are anticipated, given that the kidney and liver function as biosynthesis centers for various selenoproteins, including P‐selenoprotein and GPx [44]. In those studies, the loin Se content remained consistent across groups, which contrasts with our findings.

Overall, these findings demonstrate that selenium deposition differed among tissues under the evaluated dietary treatments. However, the mechanisms underlying these responses remain unclear and require further investigation.

### 4.5 Effect of Stress Conditions and Species Variability

Even in the absence of marked stress or conditions associated with increased oxidative challenge, dietary supplementation modulated hepatic antioxidant responses, although the magnitude of the response differed among biomarkers. However, it is plausible that different responses would be observed under stress or disease conditions.

Previous studies show that in heat‐stressed pregnant sows [15] and piglets exposed to the herbicide Diquat [54], Se consumption increased plasma antioxidant activity. Likewise, diabetic mice consuming selenized mushrooms showed hepatic and renal antioxidant responses [18].

Variability across studies likely stems from differences in animal models, Se sources and forms, environmental conditions, dosages, physiology, metabolism, and baseline Se status. For example, rats fed Se‐enriched *P. ostreatus* at 0.15 ppm showed different plasma Se dynamics than piglets on control diets, possibly reflecting differences in selenium metabolism among species [11].

### 4.6 Examples of Species‐Specific Responses

Previous studies have reported variable responses to selenium supplementation across species. For instance, calves fed with Se‐enriched *Lentinula edodes* (5 µg Se kg^−^^1^ body weight) showed a marked increase in serum selenium concentration from 38.5 to 210.7 µg L^−^^1^ [55], while horses exhibited increased plasmatic GPx activity when supplemented with 0.3 ppm selenium from selenized yeast [56].

Conversely, Koyama et al. [57] reported no plasma GPx increase in mice fed selenomethionine at 0.4 ppm; similarly, in heat‐stressed sows, supplementation with sodium selenite or selenized yeast did not alter plasma GPx activity [15]. In another study, rats with hypercholesterolemia receiving sodium selenite at 1.0 ppm showed reduced plasma NO levels [58].

Collectively, these findings suggest that plasma antioxidant responses to selenium supplementation are highly variable and may depend on factors such as species, physiological or pathological conditions, selenium source and dosage, supplementation duration, and baseline selenium status.

### 4.7 NO and MDA as Toxicity/Oxidative Stress Indicators

Monitoring NL, an indirect marker of nitric oxide (NO) metabolism, together with MDA helps assess oxidative status and the potential biological effects of foods such as Se‐enriched mushroom. NO acts as a vasodilator and is involved in inflammatory processes; when it reacts with superoxide, it forms peroxynitrite, which can damage proteins and DNA [22, 59]. Therefore, changes in NL may reflect alterations in NO metabolism and oxidative status in the liver.

When elevated, MDA, the final product of polyunsaturated fatty acid decomposition, is associated with cardiovascular, hepatic, metabolic, and neurodegenerative diseases, in addition to diabetes, Alzheimer's, Parkinson's, and cancer [60].

In the present study, the reduction in NL in the liver in piglets fed both unenriched mushroom and selenized compounds suggests a modulation of oxidative status, although no significant changes were observed in MDA levels. This indicates that, under the conditions evaluated, the dietary treatments may have influenced specific aspects of the antioxidant system without markedly affecting lipid peroxidation.

Supporting this interpretation, in mice with acetaminophen‐induced hepatic injury, supplementation with 10% *P. ostreatus* maintained MDA, GPx, and SOD activities in the liver [61].

### 4.8 Selenium Metabolism and Physiological Considerations

The data and cited literature highlight that Se ingestion impacts antioxidant biomarkers (GPx, CAT, SOD, NL, MDA), particularly in the liver. The form of Se (organic/inorganic), administration method, dietary Se content, and organism needs crucially influence metabolism and distribution. In Se depletion, the organism prioritizes uptake by the brain, reproductive organs, and endocrine glands, reducing GPx activity elsewhere; with consistently elevated Se intake, excretion (primarily urinary) increases [22]. These dynamics underscore the complex interplay among Se forms, physiological demand, and antioxidant balance.

### 4.9 Study Limitations

While this study demonstrates that dietary supplementation modulated hepatic antioxidant responses, some limitations of the experimental design should be acknowledged. The absence of a non‐selenized yeast control group limits our ability to distinguish the specific effects of selenium enrichment from the inherent bioactive properties of the yeast matrix. Likewise, although changes in antioxidant biomarkers were observed in the group receiving non‐enriched mushroom (G2), the present experimental design does not allow the specific contribution of mushroom‐derived bioactive compounds to be separated from the effects of selenium supplementation. Furthermore, because the study was not designed to evaluate interactions between selenium and mushroom constituents, no conclusions regarding synergistic effects can be drawn. Additional studies including appropriate control groups and experimental designs specifically addressing these interactions are required to clarify the relative contribution of each component.

## 5 Conclusions

In this study, we evaluated the effects of dietary supplementation with non‐enriched mushroom, selenium‐enriched mushroom, and selenized yeast on growth performance, antioxidant responses, and selenium deposition in piglets. The absence of changes in ADG, ADI, and FCR indicates that these dietary treatments were well tolerated and did not impair animal performance, supporting their safety for inclusion in piglets’ diets.

Dietary treatments influenced selenium distribution in specific tissues. Selenium concentration was higher in the loin of piglets from G2, G3, and G4 than in the control group, whereas no consistent significant differences were observed in the liver and kidney. These findings indicate that selenium deposition differed among tissues under the conditions evaluated.

Hepatic antioxidant activity was modulated by dietary treatments, although the response depended on the biomarker evaluated. GPx activity increased in all supplemented groups, whereas CAT activity increased only in piglets receiving selenium‐enriched mushroom or selenized yeast, and SOD activity increased only in the selenium‐enriched mushroom group. In contrast, NL were reduced in all supplemented groups, while MDA remained unchanged. These findings indicate that dietary supplementation modulated hepatic antioxidant responses; however, the present experimental design did not allow the relative effects of selenium enrichment and mushroom‐derived bioactive compounds to be distinguished.

Overall, this study shows that dietary supplementation with both non‐enriched and selenium‐enriched mushrooms, as well as selenized yeast, modulated antioxidant responses in piglets and influenced selenium deposition in a tissue‐dependent manner. These findings support the potential of mushrooms as functional dietary ingredients and of selenium‐enriched dietary supplements for modulating antioxidant responses, while highlighting the need for further studies to clarify the relative contribution of selenium enrichment and mushroom‐derived bioactive compounds under different physiological conditions.

## Author Contributions

**Diene France de Souza**: conceptualization, methodology, formal analysis, investigation, writing – original draft, writing – review & editing. **Gabriel Cipriano Rocha**: conceptualization, methodology, formal analysis, investigation, writing – original draft, writing – review & editing. **Dairon Pérez Fuentes and Lucas Eduardo Oliveira Porto**: methodology—selenium analysis. **Pedro Vitoriano Oliveira**: methodology—selenium analysis, resources, writing – review & editing. **Marliane de Cássia Soares da Silva**: conceptualization, methodology, resources, writing – review & editing, supervision. **Maria Catarina Megumi Kasuya**: conceptualization, resources, writing – review & editing. **Maiane de Paula Alves**: methodology, writing – review & editing. **Monique Renon Eller**: conceptualization, resources, supervision, writing – review & editing.

## Funding

This work was supported by the Coordenação de Aperfeiçoamento de Pessoal de nível Superior (CAPES), under Grant 001‐Diene France de Souza.

## Ethics Statement

This study was approved by the Ethics Committee on the Use of Production Animals—CEUAP/UFV, process number 106/2018.

## Conflicts of Interest

The authors declare no conflicts of interest.

## Use of Generative AI and AI‐Assisted Technologies in the Writing Process

During the preparation of this manuscript, the authors used large language models (LLMs) in a supportive editorial capacity. Specifically, ChatGPT 5.1 (OpenAI) and Gemini 1.5 Pro (Google) were used for translation, grammar checking, and general writing refinement. Figure 1 and the graphical abstract were created using ChatGPT, while Figure 2 was produced using Claude Sonnet 5.0 (Anthropic). All elements were created under detailed author guidance, with all scientific elements, layout decisions, and content accuracy defined and verified by the authors. Any AI‐generated material included in this manuscript was critically evaluated and validated by the authors, who assumed full responsibility for its accuracy and integrity.

## Data Availability

Data will be provided upon request.
